# Exploratory plasma proteomic analysis in a randomized crossover trial of aspirin among healthy men and women

**DOI:** 10.1371/journal.pone.0178444

**Published:** 2017-05-25

**Authors:** Xiaoliang Wang, Ali Shojaie, Yuzheng Zhang, David Shelley, Paul D. Lampe, Lisa Levy, Ulrike Peters, John D. Potter, Emily White, Johanna W. Lampe

**Affiliations:** 1 Department of Epidemiology, University of Washington, Seattle, Washington, United States of America; 2 Division of Public Health Sciences, Fred Hutchinson Cancer Research Center, Seattle, Washington, United States of America; 3 Department of Biostatistics, University of Washington, Seattle, Washington, United States of America; National Natural Science Foundation of China, CHINA

## Abstract

Long-term use of aspirin is associated with lower risk of colorectal cancer and other cancers; however, the mechanism of chemopreventive effect of aspirin is not fully understood. Animal studies suggest that COX-2, NFκB signaling and Wnt/β-catenin pathways may play a role, but no clinical trials have systematically evaluated the biological response to aspirin in healthy humans. Using a high-density antibody array, we assessed the difference in plasma protein levels after 60 days of regular dose aspirin (325 mg/day) compared to placebo in a randomized double-blinded crossover trial of 44 healthy non-smoking men and women, aged 21–45 years. The plasma proteome was analyzed on an antibody microarray with ~3,300 full-length antibodies, printed in triplicate. Moderated paired t-tests were performed on individual antibodies, and gene-set analyses were performed based on KEGG and GO pathways. Among the 3,000 antibodies analyzed, statistically significant differences in plasma protein levels were observed for nine antibodies after adjusting for false discoveries (FDR adjusted p-value<0.1). The most significant protein was succinate dehydrogenase subunit C (SDHC), a key enzyme complex of the mitochondrial tricarboxylic acid (TCA) cycle. The other statistically significant proteins (NR2F1, MSI1, MYH1, FOXO1, KHDRBS3, NFKBIE, LYZ and IKZF1) are involved in multiple pathways, including DNA base-pair repair, inflammation and oncogenic pathways. None of the 258 KEGG and 1,139 GO pathways was found to be statistically significant after FDR adjustment. This study suggests several chemopreventive mechanisms of aspirin in humans, which have previously been reported to play a role in anti- or pro-carcinogenesis in cell systems; however, larger, confirmatory studies are needed.

## Introduction

Low-dose and regular-strength aspirin use is consistently observed to be associated with reduced long-term risk of colorectal cancer (CRC) risk of adenomatous polyps, pre-cancerous lesions that increase risk of CRC [1, 2]. Benefit increases with duration of aspirin use and is associated with 34% reduction in 20-year CRC risk [1, 3]. Evidence consistently suggests that aspirin plays a role at an early stage or even before tumorigenesis [4]. Therefore, studies of aspirin involving healthy individuals may help elucidate biological responses related to the chemopreventive effects of aspirin.

The presumed main mechanism by which aspirin lowers adenomatous polyps and CRC risk is by reducing inflammatory mediators through the inhibition of cyclooxygenase-2 (COX-2) activity [5, 6] and subsequent formation of prostaglandin E2 (PGE2) [7]. Aspirin has also been shown to inhibit the oncogenic Wnt/β-catenin pathway [8] and the extracellular-signal-regulated kinase (ERK) signaling pathway [9] in colon cancer cell lines. Indirect support for these pathways from human studies comes from nested case-control studies which suggest that interactions between the use of non-steroidal anti-inflammatory drugs (NSAIDs) and polymorphisms in oncogenes in the Wnt/β-catenin signaling pathway [10] and NFкB-signaling pathway [11] modify CRC risk [12]. Recently, a genome-wide investigation of gene-environment interactions reported that the association of NSAIDs with CRC risk differed according to genetic variation at 2 SNPs [13]; these are related to genes involved in activation of the PI3K signaling pathway. Other pathways related to transcription factors, cell proliferation and apoptosis have also been suggested [14].

No human intervention trials have yet systematically explored proteomic profiling of aspirin use among human subjects. A randomized controlled trial of diclofenac among overweight individuals identified a group of inflammation-related modulators [15]; another trial suggested a variety of pathways, including cytokine activity pathways, in response to glucosamine and chondroitin supplementation [16]. These studies support the utility of proteomic profiling in characterizing responses to drugs or supplements with pleiotropic effects. We created a high density antibody array containing >3,200 different antibodies to ~2,100 different proteins that we use to interrogate plasma or other biological samples for cellular activation status including proteins involved in apoptosis, proliferation, angiogenesis, immune cell activity/infiltration, and metabolism, etc. Many of the antibodies are to secreted proteins such as cytokines and growth factors including 21 proteins with insulin in their names. We have used these arrays to find biomarkers of ovarian [17, 18], breast [19], pancreas [20, 21] and colon [22] cancer and used the values derived to find pathways important in obesity [23], supplement usage [16], anti-apoptotic cell survival signaling pathways [22], and incisional hernia [24]. The objective of this study was to explore potential mechanisms relevant to the effects of aspirin through proteomic analysis in healthy participants in a randomized trial of aspirin, with a focus on proteins that are related to cancer development.

## Materials and methods

### Study design

The Aspirin and the Biology of the Colon (ABC) study was a randomized, double-blinded, placebo-controlled, crossover trial [25]. During each intervention period, participants took 325 mg aspirin or a visually identical placebo orally each day for 60 days, with a 3-month washout period between the treatment periods. Study activities, including participant interviews and blood draws, were conducted at the Fred Hutchinson Cancer Research Center (Fred Hutch) Prevention Center Research Clinic. The study procedures were approved by the Fred Hutch Institutional Review Board; informed, written consent was obtained from all participants prior to participation in the study.

### Study participants

Details of the study and the study population have been described previously [25, 26] and are summarized in S1 Fig. Briefly, healthy men and women, aged 21 to 45 years, were recruited from participants from the greater Seattle area who completed a cross-sectional study of diet and aspirin metabolism between June 2003 and March 2007 [27]. Individuals were excluded if they had: a medical history of gastrointestinal, hepatic, or renal disorders; family history of familial adenomatous polyposis or Lynch syndrome; known intolerance to aspirin or other NSAIDs; weight change greater than 4.5 kg within the past year; current use of prescription medication (including oral contraceptives) or over-the-counter medications; alcohol intake >2 drinks/day; were pregnant or lactating; or were planning to move out of the greater Seattle area within the 12 months of the study period. Given that an aim of the parent study was to determine whether genetic variation in *UGT1A6* influenced response to aspirin [25], participants who met these criteria were further selected based on *UGT1A6* genotypes (rs2070959 and rs1105879) so that all subjects with a *2/*2 genotype and sex-matched participants with a *1/*1 genotype were invited to participate. Additionally, two participants with a *2/*4 genotype were also included and randomized. Clinical measurements were also assessed and participants with abnormal laboratory values were excluded from participation. A total of 55 healthy men and women were recruited into the trial, randomly assigned, blocked on sex and *UGT1A6* genotype, to the order of receiving aspirin or placebo. Forty-four participants completed both intervention periods. The reasons participants dropped out were not related to either intervention or placebo period.

### Data collection

Demographics and medical history were obtained through questionnaires at the time of recruitment, including age, ethnicity, previous smoking habits, dietary supplement use, alcohol intake, history of weight change, and general health. Twelve-hour fasting morning blood samples were drawn on day -5 and day 55 of the first intervention period, and days 1 and 55 of the second intervention period. The pre-intervention blood samples were tested for liver and kidney function, and post-intervention blood samples were used for research purposes. Blood samples were collected in EDTA-containing vacutainer tubes; plasma was aliquoted into cryovials and stored at -80°C until analysis.

### Proteomics analyses

Plasma samples were analyzed on a customized antibody array populated with ~3,300 full-length antibodies, printed in triplicate on a single microarray according to published methods [20–24, 28]. Briefly, each sample (200 μg) was combined with the same amount of a Cy3 labeled “reference” pool (from 5 healthy men and 5 women) of albumin and IgG-depleted plasma, placed on the array and Cy3 and Cy5 signals determined. The log2-transformed Cy5/Cy3 ratio, noted as M value, determined the relative concentration of protein compared to reference. For quality-control purposes, triplicate antibodies with coefficients of variation >10% were removed, and experimental variation was normalized using within-array print-tip loess normalization and between-array quartile normalization [29]. The median for each antibody was taken from triplicates as the summary measure.

The two samples from the same person collected at the termination of the two periods (aspirin and placebo) were analyzed in the same batch with the order of treatment periods randomized. Sex and genotypes were randomly distributed across batches. Additional possible batch effects were checked by principal component analysis (data not shown).

Previous analyses showed that coefficients of variation, for triplicates, for >85% antibodies on the array were less than 10% [18, 28, 30, 31]. Intra-class correlation (R_1_) for triplicates was also used to evaluate the reliability of triplicates in this study [32]. 92.5% of the antibodies had at least moderate correlation among triplicates (R_1_>0.5), and 83.5% antibodies had strong correlations among triplicates (R_1_>0.7), suggesting reliable measurements of plasma protein levels. Antibodies were also highly correlated between quality-control duplicate samples that were blinded to the lab analysist.

### Statistical analysis

Antibodies with more than 30% missing values across the arrays were excluded from further analysis. Remaining missing data were imputed using the local least squares imputation method, which replaces a target protein that has missing values with a linear combination of 10 similar proteins, chosen by k-nearest neighbors based on Pearson correlation coefficients [33]. Of ~3,300 antibodies on the array, 3,000 proteins were available for statistical analyses and complete data were available on all 44 participants after imputation. Moderated paired t-tests [34] were performed for individual proteins to determine statistically significant differences between aspirin and placebo treatments. Adjustment for potential confounders, including batch effect, sample positions, and covariate effects of sex and *UGT1A6* genotype was carried out using a mixed linear regression model. The proteins were then ranked on the basis of p-values adjusted by Benjamini-Hochberg false discovery rate (FDR) correction, at a significance level of 0.1 [34]. Both the moderated t-tests and FDR corrections were performed using the R LIMMA package [29].

Pathway analyses were also carried out [35], using gene sets in Kyoto Encyclopedia of Genes and Genomes (KEGG) and the Gene Ontology (GO). KEGG gene sets were obtained via REST server to KEGG, and GO gene sets were obtained from MSigDB (http://www.broadinstitute.org/gsea/msigdb/index.jsp). Simulation analyses were performed to compare several gene-set analytical tools [35–37]. The Significance Analysis of Functional Categories (SAFE) framework [35] outperformed the other methods in relation to sensitivity and specificity, and was used in further analysis. Gene sets with fewer than 3 genes were excluded from the analysis. The enriched gene sets were ranked on the basis of adjusted p-values, with an FDR significance level of 0.1.

## Results

The demographic characteristics of the 44 study participants are summarized in Table 1. The study population was predominantly Caucasian, and approximately half of the participants were overweight or obese (BMI≥25 kg/m^2^).

**Table 1:** Demographic characteristics of 44 participants.

Characteristics	N (%)
Age, Mean(SD)	30.43 (5.97)
Sex	
Male	20 (45.5)
Female	24 (54.5)
BMI, kg/m^2^	
Normal (<25)	23 (52.3)
Overweight or obese (≥25)	21 (47.7)
Race/Ethnicity	
Caucasian	33 (75.0)
African-American	1 (2.3)
Asian	5 (11.4)
Other	5 (11.4)

Among the 3,000 proteins tested, nine were statistically different between the aspirin and placebo periods after FDR correction (adjusted p-value<0.1) (Table 2), including energy convertors, hormone receptors, transcriptional factors, and RNA- and DNA-binding proteins. Among these nine proteins, six (MYH1, FOXO1, KHDRBS3, NFKBIE, LYZ and IKZF1) had a higher expression level on aspirin than placebo, whereas the other three (SDHC, NR2F1 and MSI1) had a lower expression level on aspirin than placebo. The most significant protein was succinate dehydrogenase subunit C (SDHC), with an average 34% lower expression level on aspirin than placebo (p-value = 4.47×10^−05^; FDR-adjusted p-value = 0.06). The next most significant was myosin-1 (MYH1). The expression level of MYH1 on aspirin treatment was 62% higher on average than that on placebo (p-value = 6.83×10^−05^; FDR-adjusted p-value = 0.06). The largest decrease was observed in a nuclear hormone receptor NR2F1 with 60% lower expression level on aspirin than placebo. NF-κB inhibitor epsilon (NFKBIE) had one of the largest increases: the expression level was 63% higher on aspirin than placebo. Because the study was conducted as a randomized crossover study, each participant served as their own control; predictably, adjusting for batch effects, sample positions, order of aspirin and placebo period, and other covariates did not change test results.

**Table 2:** Proteins that differed significantly between aspirin and placebo periods with false discovery rate (FDR) <0.10.

Symbol	Function^a^	Missing%^b^	Average expression^c^		Effect size^d^	Fold change^e^	p-value^f^	Adjusted p-value^g^
			aspirin	placebo				
SDHC	Conservative effector of mitochondrial Krebs cycle and respiratory chain	20.5	-0.259	-0.112	-0.594	0.662	4.47×10^−05^	0.058
MYH1	Energy convertor in base excision repair (BER) pathway	18.2	-0.262	-0.454	0.698	1.622	6.83×10^−05^	0.058
NR2F1	Nuclear hormone receptor and transcriptional regulator	11.4	-0.371	-0.265	-0.755	0.592	6.94×10^−05^	0.058
FOXO1	Transcription factor in carbohydrates metabolism and Akt-mTOR signaling pathway	17.0	1.003	0.845	0.520	1.434	7.72×10^−05^	0.058
KHDRBS3	RNA-binding protein regulating pre-mRNA splicing, signaling and cell cycle control	27.3	0.096	-0.028	0.400	1.320	2.07×10^−04^	0.087
NFKBIE	NF-κB inhibitor epsilon	23.9	0.530	0.374	0.709	1.634	2.15×10^−04^	0.087
LYZ	Lysozyme, antimicrobial enzyme	26.1	0.004	-0.147	0.689	1.612	2.29×10^−04^	0.087
MSI1	RNA-binding protein, posttranscriptional regulator of proliferative activity	26.1	-0.351	-0.176	-0.487	0.714	2.32×10^−04^	0.087
IKZF1	Transcription factor of zinc-finger DNA-binding and lymphocyte differentiation regulator	4.5	-0.310	-0.426	0.542	1.456	2.67×10^−04^	0.089

^a^ Information pertaining to encoded protein function was derived from PubMed Gene unless otherwise noted.

^b^ Missing% is the proportion of samples with missing values on this protein among all 88 samples.

^c^ Average expression level was presented in median M values for each protein.

^d^ Effect size was the mean difference of M values between two treatment periods standardized by standard deviation of average expression in placebo period.

^e^ Fold-change was the standardized ratio between median M values of aspirin and placebo treatment. A fold change >1 indicated greater antibody expression after aspirin treatment compared to placebo; a fold change <1 indicated lower expression after aspirin treatment.

^f^ P-values were obtained from mixed linear regression model, adjusted for batch effect, sample position, gender and genotype.

^g^ P-values were adjusted for false discovery rate using Benjamini-Horchberg procedure.

Analyses of differences in individual protein expression levels were also undertaken stratified by sex (S1 Table). For the nine proteins that were statistically different in the overall analysis, the direction of effect (increased or decreased) was consistent across men and women; however, none of the within-group changes in protein levels was statistically significant after FDR correction, probably due to reduced sample size in subgroup analyses.

In gene-set analyses using the SAFE framework, a total of 257 KEGG pathways and 1139 GO pathways were tested. Among them, 21 KEGG and 63 GO pathways were statistically significantly different on aspirin than placebo (p-value<0.05); however, none of these gene categories reached statistical significance after FDR correction at significance level of 0.1 (S2 Table).

## Discussion

In this placebo-controlled randomized crossover trial among healthy individuals, plasma levels of nine of the total of ~3,000 antibodies were significantly different between 60-day regular-dose aspirin and placebo, after adjustment for FDR at 0.1. Among these nine proteins, six had higher expression levels, and three were lower on aspirin than placebo. These proteins play important roles in various pathways, including the mitochondrial Kreb’s cycle, DNA base-pair repair, and inflammation. However, when correcting for multiple comparisons, we did not identify overall pathways that were statistically significantly different between aspirin and placebo.

The protein with the most significant difference between treatments was SDHC; plasma levels were lower after aspirin treatment. SDHC is a subunit of succinate dehydrogenase (SDH), a key enzyme complex of the mitochondrial tricarboxylic acid (TCA) cycle, which oxidizes succinate to fumarate [38]. As one part of SDH (also called complex II), it also facilitates transfer of electrons to coenzyme Q (ubiquinone) [39]. Aspirin has been shown to interfere with mitochondrial function [40], as well as inhibit the activity of SDH in rats [41]. Further, repeated mild inhibition of oxidative phosphorylation via inhibition of SDH protects against the decrease in ATP that usually accompanies severe hypoxia and thus can act as neuroprotection [42]. Treatment with aspirin has also been shown *in vivo* to slow down the decline of intracellular ATP by this mechanism of inhibiting SDH [43] and therefore to protect against hypoxia, a common hallmark of tumors that promotes metabolic adaptations and angiogenesis [44]. Furthermore, accumulation of succinate, due to reduced efficiency of SDHC, results in the stabilization of HIF1-α, the degradation of which is promoted by the oncometabolite (R)-2-hydroxyglutarate [45]. Metabolomic analysis in the present study has shown that plasma concentrations of 2-hydroxyglutarate decreased after aspirin treatment in both men and women (p = 0.005) [26]. It is relevant that plasma concentration of HIF1-α was lower after aspirin treatment among carriers of wild UGT1A6*1/*1 genotype (Data not shown; p = 6.2×10^−05^; adjusted p-value = 0.186), but not among carriers of UGT1A6*2/*2 genotype, suggesting that the genotypes of *UGT1A6*, which encodes a UDP-glucuronosyltransferase that participates in glucuronidation of aspirin [46], may modulate the effect of aspirin on downstream metabolic functions. In summary, results from our proteomic analyses, as well as those from metabolomic analyses support a possible additional mechanism for aspirin in cancer prevention.

The expression level of MYH1 was also statistically significantly higher by 62% on aspirin than placebo. As a member of the human homologue of the base excision repair (BER) gene, MYH1 is a DNA glycosylase that removes adenine mispaired with 8-hydroxyguanine from DNA and protects against oxidative DNA damage [47, 48]. Inherited variants in *MYH* that cause reduced enzyme function have been associated with significantly increased risk of familial and sporadic CRC in observational studies [49–52]. In addition, the prevalence of low-frequency microsatellite instability (MSI) has been found to be higher among *MYH* mutation carriers [51, 53], suggesting possible interaction between the BER and MSI pathways. However, most previous observational studies assessed the association between germline mutations of *MYH* and CRC risk, whereas our study directly measured the plasma level of MYH1. Our findings suggest that environmental factors, such as aspirin, may also have an effect on enzyme levels, regardless of genetic background.

Similarly, NFKBIE was 63% higher on average on aspirin than placebo. NFKBIE is involved in the NF-κB signaling pathway. After cellular stimulation, NFKBIE is highly induced to bind the NF-κB dimer, and provides negative feedback regulation that inhibits NF-κB DNA-binding activity and prevents its nuclear accumulation [54]. As the NF-κB pathway plays an important role in chronic inflammation and tumor promotion, reduction of NF-κB activity is critical in inhibiting the production of pro-inflammatory cytokines [55, 56]. In an observational study among 315 chronic lymphocytic leukemia (CLL) patients, targeted deep sequencing of 18 core complex genes within the NF-κB pathway found that the most frequently mutated genes was *NFKBIE*; further screening revealed that truncated NFKBIE predominated in patients with poor prognosis [57]. Similarly, exome sequencing has also suggested that NFKBIE was highly mutated among melanoma patients [58]. Aspirin has been found to inhibit IκB kinase (IKK) β, which inhibits NF-κB inhibitors by phosphorylation [59]. In a nested case-control study, polymorphisms in IκBKβ were associated with lower CRC risk and the association was stronger among current NSAID users [11]. Findings from our study are among the first in humans to suggest a biologic interaction between aspirin and NFKBIE, thus providing further support for the likelihood that the NF-κB signaling pathway is involved in one of the mechanisms of action of aspirin.

Among the other significant proteins, aspirin treatment was associated with 29% lower level of Musashi1 (MSI1), a neural RNA-binding protein. A previous study among colon cancer patients has shown that overexpression of MSI1 in colon cancer lesions, compared to paired normal colonic mucosa, was associated with poorer metastasis-free survival and poorer overall survival [60]. A variety of other potential mechanisms have also been suggested by our findings, including those involving NR2F1 as a nuclear hormone receptor, Forkhead Box O1 (FOXO1) in Akt-mTOR signaling pathway, and KHDRBS3 in RNA-binding and cell-cycle control. Several of these have limited evidence for a relationship with aspirin in humans, probably due to the fact that most previous human studies have focused on candidate genes, proteins, or pathways. Randomized controlled trials of aspirin conducted among patients with cardiovascular disease found statistically significant reductions in circulating concentrations of high-sensitivity C-reactive protein (CRP), tumor necrosis factor-alpha (TNF-α), interleukin-6 (IL-6), and thromboxane B2 (TXB2) [61–63].

Compared to previous human studies of aspirin or other NSAIDs, our study has the strength that our antibody microarray has a high coverage of the proteome (2,100 proteins from 3,000 antibodies), which allowed for more complex proteomic profiling of the impact of aspirin. Most of the proteins that differed significantly between aspirin and placebo treatment are involved in various pathways associated with carcinogenesis, illustrating the potential range of biologic effects of aspirin *in vivo*. Secondly, most of the previous studies were focused on mechanisms inhibited by aspirin in tumor cells or in patients with a focus on tumor progression; ours is among the first to directly evaluate the effects of aspirin among healthy individuals. Therefore, we had the opportunity to identify, agnostically, proteins and mechanisms that are promoted by aspirin treatment; this, nonetheless, remains relevant to understanding how aspirin prevents tumor progression. In addition, the crossover design allowed participants to serve as their own control, which minimized unmeasured inter-individual variability. Because the samples from the same participant were randomly ordered and analyzed within the same batch, additional adjustment for batch effects and sample positions did not change the results. There was also a 3-month wash-out period between treatments (longer than the usual 60-day periods); this minimizes carry-over effects.

There are also some limitations. First, our analysis was primarily designed to examine the signaling effects of aspirin and has no specific hypothesis or protein to validate. Subsequent steps would involve testing on another set of subjects who took aspirin or placebo but these samples are not available to us at this time. Second, the duration of treatment was two months, and this may not characterize the long-term effects of aspirin use in cancer prevention. Thirdly, our sample size was relatively small and lacked substantial as plasma protein levels were not the main outcome of the intervention. Effect sizes were also small which may reflect the fact that all the participants were generally healthy and therefore would have had relatively low expression of pro-inflammatory proteins; this, in turn would leave less room for change with aspirin intervention. Furthermore, the expression levels of proteins are context-specific: our findings using plasma may differ from those in other tissues, such as colon mucosa. Six of the nine proteins typically are found in the nucleus, so their presence in plasma might result from cellular leakage/damage or exosome formation. In support of the latter, aspirin has been reported to affect the content of platelet-derived exosomes [64]. Lastly, we used a liberal significance level of 0.1 for FDR adjustment. Although a higher threshold was used to identify more possible candidates, it might also lead to more false positive results. Therefore, further investigations of these identified proteins are needed.

To our knowledge, this is the first randomized trial to systematically evaluate the effect of aspirin on plasma protein profiles in healthy men and women. We identified several proteins that differed significantly between aspirin and placebo, some of which have been previously reported as playing a role in inflammation and carcinogenesis. The involvement of various biologic pathways suggests that the chemopreventive mechanisms of aspirin in humans are complex. Larger, confirmatory studies with a longer period of aspirin exposure are needed.

## Supporting information

S1 Data File Participant information. (XLSX)

S2 Data File Microarray data. (XLSX)

S1 Fig Flow chart of participant enrollment and study design. (DOCX)

S1 Table Plasma level differences in the expression of the nine significant proteins, stratified by sex. (DOCX)

S2 Table List of gene sets with unadjusted p-values <0.05. (DOCX)
