# Supplementary figures and images for: Exploratory plasma proteomic analysis in a randomized crossover trial of aspirin among healthy men and women
Source: PLoS One. 2017 May 25;12(5):e0178444. doi: 10.1371/journal.pone.0178444 (PMC5444835; doi:10.1371/journal.pone.0178444)

**S1 Fig. Flow chart of participant enrollment and study design**

**
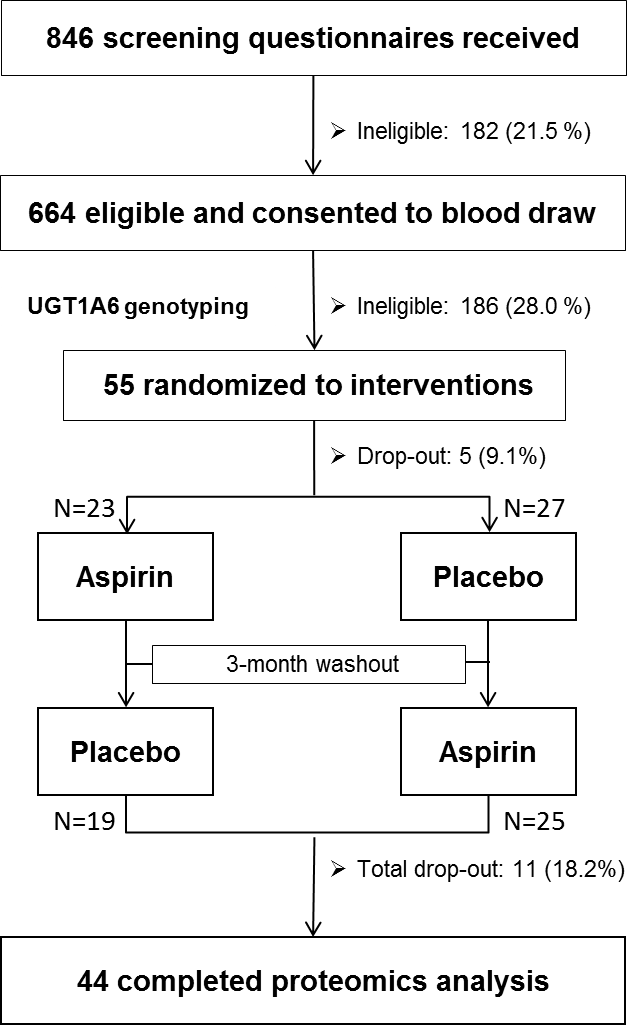
**

Supplement: S1 Fig — (DOCX) [file pone.0178444.s003.docx]
