# Supplementary material for: Exploratory plasma proteomic analysis in a randomized crossover trial of aspirin among healthy men and women
Source: PLoS One. 2017 May 25;12(5):e0178444. doi: 10.1371/journal.pone.0178444 (PMC5444835; doi:10.1371/journal.pone.0178444)
Supplement: S1 Table — (DOCX) [file pone.0178444.s004.docx]

**S1 Table. Plasma level differences in the expression of the nine significant proteins, stratified by sex.**

|  | **Male (N=20)** | | | | | |  | **Female (N=24)** | | | | | |
| --- | --- | --- | --- | --- | --- | --- | --- | --- | --- | --- | --- | --- | --- |
| **Gene name** | **Average expression**^a^ | | **Effect size**^b^ | **Fold change**^c^ | **p-value** | **Adjusted**  **p-value**^d^ |  | **Average expression**^a^ | | **Effect size**^b^ | **Fold change**^c^ | **p-value** | **Adjusted**  **p-value**^d^ |
|  | **aspirin** | **placebo** |  |  |  |  |  | **aspirin** | **placebo** |  |  |  |  |
| SDHC | -0.189 | -0.076 | -0.432 | 0.741 | 7.19×10^-03^ | 0.73 |  | -0.318 | -0.142 | -0.743 | 0.597 | 1.77×10^-03^ | 0.36 |
| MYH1 | -0.295 | -0.387 | 0.421 | 1.339 | 9.21×10^-02^ | 0.85 |  | -0.235 | -0.510 | 0.894 | 1.859 | 1.51×10^-04^ | 0.23 |
| NR2F1 | -0.385 | -0.292 | -0.615 | 0.653 | 1.61×10^-02^ | 0.73 |  | -0.360 | -0.242 | -0.901 | 0.536 | 2.38×10^-03^ | 0.36 |
| FOXO1 | 0.986 | 0.821 | 0.660 | 1.580 | 2.94×10^-03^ | 0.73 |  | 1.017 | 0.864 | 0.440 | 1.356 | 7.23×10^-03^ | 0.45 |
| KHDRBS3 | 0.064 | -0.001 | 0.192 | 1.142 | 1.44×10^-01^ | 0.85 |  | 0.121 | -0.050 | 0.604 | 1.520 | 3.23×10^-04^ | 0.23 |
| NFKBIE | 0.471 | 0.365 | 0.439 | 1.355 | 1.21×10^-02^ | 0.73 |  | 0.579 | 0.381 | 0.958 | 1.943 | 3.68×10^-03^ | 0.37 |
| LYZ | 0.023 | -0.152 | 0.749 | 1.680 | 1.65×10^-02^ | 0.73 |  | -0.013 | -0.143 | 0.620 | 1.537 | 2.16×10^-03^ | 0.36 |
| MSI1 | -0.292 | -0.113 | -0.478 | 0.718 | 1.48×10^-02^ | 0.73 |  | -0.400 | -0.229 | -0.497 | 0.709 | 4.69×10^-03^ | 0.40 |
| IKZF1 | -0.289 | -0.438 | 0.656 | 1.575 | 1.43×10^-03^ | 0.73 |  | -0.335 | -0.415 | 0.419 | 1.337 | 5.10×10^-02^ | 0.65 |

^a^ Average expression level was presented in median M values for each protein.

^b^ Effect size was the mean difference of M values between two treatment periods standardized by standard deviation of average expression in placebo period .

^c^ Fold-change was the standardized ratio between median M values of aspirin and placebo treatment. A fold-change >1 indicated greater antibody expression after aspirin treatment compared to placebo; a fold change <1 indicated lower expression after aspirin treatment.

^d^ P-values were adjusted for false discovery rate using Benjamini-Horchberg procedure.
