# Supplementary material for: Exploratory plasma proteomic analysis in a randomized crossover trial of aspirin among healthy men and women
Source: PLoS One. 2017 May 25;12(5):e0178444. doi: 10.1371/journal.pone.0178444 (PMC5444835; doi:10.1371/journal.pone.0178444)
Supplement: S2 Table — (DOCX) [file pone.0178444.s005.docx]

**S2 Table. List of gene sets with unadjusted p-values <0.05.**

| **Database** | **Gene set name** | **Size**^a^ | **p value** | **Adjusted p-value**^b^ |
| --- | --- | --- | --- | --- |
| **KEGG** | Pertussis | 55 | 0.001 | 0.257 |
|  | Retinol metabolism | 6 | 0.003 | 0.386 |
|  | Salmonella infection | 43 | 0.007 | 0.551 |
|  | Cytosolic DNA-sensing pathway | 21 | 0.013 | 0.551 |
|  | Propanoate metabolism | 3 | 0.013 | 0.551 |
|  | Rap1 signaling pathway | 150 | 0.014 | 0.551 |
|  | Spliceosome | 12 | 0.015 | 0.551 |
|  | Prion diseases | 35 | 0.019 | 0.570 |
|  | Phagosome | 55 | 0.026 | 0.570 |
|  | Glyoxylate and dicarboxylate metabolism | 7 | 0.026 | 0.570 |
|  | NOD-like receptor signaling pathway | 35 | 0.030 | 0.570 |
|  | Rheumatoid arthritis | 103 | 0.032 | 0.570 |
|  | Bile secretion | 9 | 0.034 | 0.570 |
|  | Amphetamine addiction | 13 | 0.036 | 0.570 |
|  | Arachidonic acid metabolism | 8 | 0.037 | 0.570 |
|  | RIG-I-like receptor signaling pathway | 24 | 0.040 | 0.570 |
|  | Herpes simplex infection | 82 | 0.044 | 0.570 |
|  | Vibrio cholerae infection | 9 | 0.046 | 0.570 |
|  | Gastric acid secretion | 12 | 0.047 | 0.570 |
|  | Cytokine-cytokine receptor interaction | 288 | 0.049 | 0.570 |
|  | Glutathione metabolism | 9 | 0.049 | 0.570 |
| **GO** | Oxidoreductase activity | 5 | 0.003 | 0.616 |
|  | Ion homeostasis | 89 | 0.005 | 0.616 |
|  | Cellular cation homeostasis | 83 | 0.005 | 0.616 |
|  | Cation homeostasis | 83 | 0.005 | 0.616 |
|  | MAPKkk cascade | 41 | 0.005 | 0.616 |
|  | Lipid binding | 37 | 0.005 | 0.616 |
|  | Activation of MAPK activity | 16 | 0.005 | 0.616 |
|  | Synaptic transmission | 22 | 0.006 | 0.616 |
|  | Negative regulation of response to stimulus | 10 | 0.006 | 0.616 |
|  | Cellular homeostasis | 96 | 0.007 | 0.616 |
|  | Oxidoreductase activity | 53 | 0.007 | 0.616 |
|  | Negative regulation of translation | 18 | 0.007 | 0.616 |
|  | Apoptotic nuclear changes | 7 | 0.009 | 0.616 |
|  | Nuclear organization and biogenesis | 7 | 0.009 | 0.616 |
|  | Transmission of nerve impulse | 25 | 0.010 | 0.616 |
|  | Isomerase activity | 8 | 0.010 | 0.616 |
|  | Transcription cofactor activity | 48 | 0.011 | 0.616 |
|  | Growth factor binding | 25 | 0.011 | 0.616 |
|  | Contractile fiber | 6 | 0.011 | 0.616 |
|  | Contractile fiber (part) | 6 | 0.011 | 0.616 |
|  | Response to chemical stimulus | 186 | 0.012 | 0.616 |
|  | Homeostatic process | 126 | 0.012 | 0.616 |
|  | Calcium ion binding | 38 | 0.012 | 0.616 |
|  | Regulation of cytokine biosynthetic process | 18 | 0.012 | 0.616 |
|  | Regulation of gene expression (epigenetic) | 4 | 0.013 | 0.641 |
|  | Chemical homeostasis | 102 | 0.015 | 0.655 |
|  | Brush border | 3 | 0.015 | 0.655 |
|  | Secondary metabolic process | 4 | 0.016 | 0.655 |
|  | Pigment biosynthetic process | 4 | 0.016 | 0.655 |
|  | Pigment metabolic process | 4 | 0.016 | 0.655 |
|  | Protease inhibitor activity | 23 | 0.017 | 0.655 |
|  | Tube morphogenesis | 8 | 0.017 | 0.655 |
|  | Cofactor biosynthetic process | 3 | 0.018 | 0.666 |
|  | Feeding behavior | 3 | 0.019 | 0.666 |
|  | Cysteine type endopeptidase activity | 15 | 0.020 | 0.666 |
|  | Anion transport | 5 | 0.020 | 0.666 |
|  | Late endosome | 5 | 0.020 | 0.666 |
|  | Cofactor metabolic process | 7 | 0.025 | 0.794 |
|  | External side of plasma membrane | 9 | 0.026 | 0.794 |
|  | Negative regulation of cellular biosynthetic process | 28 | 0.027 | 0.794 |
|  | Negative regulation of biosynthetic process | 28 | 0.027 | 0.794 |
|  | Transmembrane receptor activity | 121 | 0.029 | 0.794 |
|  | Hormone binding | 13 | 0.029 | 0.794 |
|  | Positive regulation of MAP kinase activity | 38 | 0.030 | 0.804 |
|  | Amino acid and derivative metabolic process | 13 | 0.032 | 0.806 |
|  | Behavior | 112 | 0.033 | 0.806 |
|  | Regulation of MAP kinase activity | 40 | 0.034 | 0.806 |
|  | Smooth muscle contraction | 5 | 0.034 | 0.806 |
|  | Structural constituent of muscle | 3 | 0.034 | 0.806 |
|  | Myofibril | 5 | 0.035 | 0.814 |
|  | Neuron projection | 5 | 0.037 | 0.828 |
|  | Transcription factor binding | 79 | 0.039 | 0.828 |
|  | Cellular component disassembly | 9 | 0.039 | 0.828 |
|  | Intramolecular oxidoreductase activity | 7 | 0.040 | 0.828 |
|  | Exopeptidase activity | 7 | 0.041 | 0.828 |
|  | Regulation of muscle contraction | 4 | 0.041 | 0.828 |
|  | Transmembrane receptor protein tyrosine kinase activity | 50 | 0.042 | 0.835 |
|  | Transmembrane receptor protein kinase activity | 51 | 0.044 | 0.860 |
|  | Cellular biosynthetic process | 92 | 0.046 | 0.865 |
|  | Negative regulation of cytokine biosynthetic process | 13 | 0.047 | 0.865 |
|  | Cortical actin cytoskeleton | 4 | 0.047 | 0.865 |
|  | Transcription corepressor activity | 24 | 0.048 | 0.865 |

^a^ Size indicated the number of genes that had corresponding proteins tested in the analysis.

^b^ P-values were adjusted for false discovery rate using Benjamini-Horchberg procedure.
